# A retrospective analysis of factors influencing the success of autotransplanted posterior teeth

**DOI:** 10.1186/s40510-015-0112-y

**Published:** 2015-11-23

**Authors:** Mirco F. Ronchetti, Silvio Valdec, Nikolaos Pandis, Michael Locher, Hubertus van Waes

**Affiliations:** Clinic of Orthodontics and Paediatric Dentistry, Center of Dental Medicine, Faculty of Medicine, University of Zürich, Zürich, Switzerland; Clinic of Oral Surgery, Center of Dental Medicine, Faculty of Medicine, University of Zürich, Zürich, Switzerland; Department of Orthodontics and Dentofacial Orthopedics, Dental School/Medical Faculty, University of Bern, Bern, Switzerland

**Keywords:** Tooth transplantation, Survival, Success factors

## Abstract

**Background:**

Survival and success rates of tooth transplantations even after long follow-up periods have been shown to be very high. Nevertheless, it is important to analyse factors potentially influencing these rates. The aim of this study was to assess the influence on success of potential factors.

**Methods:**

The research was based on a retrospective analysis of clinical and radiological data from a sample of 59 subjects (75 transplanted teeth). The follow-up period varied from 0.44 to 12.28 years (mean 3.95 years). Success rates were calculated and depicted with Kaplan-Meier plots. Log-rank tests were used to analyse the effect of root development stage, apex width, the use of enamel matrix proteins or the surgeon on success of transplantations.

**Results:**

Results for success of premolar transplantations were comparable with already published data, while molars performed worse than shown in other studies. The surgeon performing the transplantation (*p* = 0.001) and tooth type (*p* ≤ 0.001) were significantly associated with transplantation success. Use of enamel matrix proteins (*p* = 0.10), root development stage (*p* = 0.13), the recipient area (*p* = 0.48) and apex width (*p* = 0.59) were not significantly associated with success.

**Conclusions:**

Molar transplantations were not as successful as premolar transplantations; however, success rates varied greatly depending on the surgeon’s experience. The use of enamel matrix proteins as well as root development stage, the recipient area and apex width did not show significant associations with success of tooth transplantations.

## Background

The first documented case report of a homoplastic tooth transplantation was described by Fauchard in 1728 in his book *Le Chirurgien Dentiste* [1]. This procedure of tooth autotransplantation is defined as the transfer of a donor tooth to a recipient area in the same individual with the latter being either an extraction site or a surgically prepared socket. Starting from the 1970s and the publications of Slagsvold and Bjercke [2–4] and later with the studies of Andreasen and Paulsen [5–8], the autotransplantation of premolars has become a well-accepted and reliable procedure to replace either missing incisors or premolars. Survival of transplanted premolars has been shown to be high even decades after the surgery. Czochrowska showed a 90 % survival and a 79 % success rate after a mean observation period of 26.4 years post transplantation, indicating that some transplants were kept in situ over long periods of time despite clinical or radiographic signs of failure (no success). Autotransplantation also compared favourably with other procedures for replacement of missing teeth. Furthermore, when comparing clinical parameters of transplanted to non-transplanted teeth, no clinical or radiographic difference except for pulp obliteration was evident [9]. The advantage of autotransplantation compared to osseointegrated implants is the capacity of transplants for functional adaptation, potentially continuous eruption and therefore superior ridge preservation [7–11]. In addition, transplantation of molars, if well selected, has been shown by several authors to be a good treatment option for missing teeth with success ranging from 79 to 100 % [12–14].

Prior studies analysing transplantations have claimed three fourths root length as being the ideal development stage for transplantation [5, 15].

Treatment with enamel matrix proteins like Emdogain® (Biora, Malmö, Sweden; incorporated into Straumann Biologic Division since 2004) has been shown to exhibit an activating effect on periodontal cells [16–20]. Positive effects were also shown for the treatment of infrabony defects [21] as well as for prevention of ankyloses or root resorptions after intentional transplantation or treatment of avulsed teeth [22, 23]. Those findings however have been questioned by other investigators [24–26].

Although the published findings indicate very high success (tooth in situ without any clinical or radiographic sign of failure) and survival rates (tooth in situ with or without presence of clinical or radiographic signs of failure like ankylosis, periodontal issues, root resorption or crown/root ratio >1), it is important to identify all factors determining success and survival rates of tooth transplantations. These factors are the key to higher clinical success and to prevention of unnecessary failures.

The aim of this study was to record success rates of tooth transplantations and to examine parameters which may be associated with success.

## Methods

This research project was reviewed and approved by the ethics committee of the canton of Zurich, Switzerland.

All consecutive patients from the dental clinic of the University of Zürich who had undergone one or more tooth transplantations were included in order to minimize selection bias. The search was performed by a computer specialist on the patients’ database system, where all transplantations had been registered. The database was introduced in January 2000, and all registered transplantation cases up to 31 December 2012 were included. Before the year 2000, no complete patient database was available.

The following information was extracted from the retrieved files: date of birth; date of transplantation; age at transplantation; numeric number of the transplanted tooth; recipient area; root length in millimetres (measured on single X-rays) and development stage at transplantation according to Moorrees et al. [27]; apex width in millimetres grouped into ≥2 or <2 mm; post treatment development, namely root canal obliteration, root resorption, ankylosis and endodontic issues with subsequent root canal treatment including the dates of these events; and surgeon performing the surgery. Transplantations were performed by three surgeons with different levels of experience. Surgeon 1 is the clinical director of the Clinic of Paediatric Dentistry with long experience in transplantations; surgeon 2 was a postgraduate student at the Clinic of Paediatric Dentistry; and surgeon 3 was the former director of the Clinic of Oral Surgery with broad surgical experience.

The individual success period of each transplanted tooth was defined as the time period from the transplantation to the latest exam appointment where the tooth was in situ with no clinical signs of failure. Progressive root resorption, periodontal problems, ankylosis and crown to root ratio greater than 1.0 were considered to be failures [9, 28], whereas endodontic treatment was not rated as a failure.

The radiographic evaluation was carried out by two researchers independently. Results were subsequently discussed until consensus was reached.

Data were coded in Excel (version 14.0.6112.5000, Redmond, WA, USA) and analysed using STATA statistical software (version 14.0, STATA Corp, College Station, TX, USA). Descriptive statistics such as mean, median, standard deviation and absolute and relative frequencies were computed. Kaplan-Meier plots were produced, and association between success and root development stages, application of enamel matrix proteins, apex width, recipient area and surgeon were examined using the log-rank test. The root development stage according to Moorrees [27] was grouped into shorter than three fourths (corresponding to Moorrees’ levels 1 and 2), three fourths (corresponding to Moorrees’ level 3) and longer than three fourths (corresponding to Moorrees’ levels 4–6) while apex width at transplantation was divided into *x* < 2 mm and *x* ≥ 2 mm. Results of statistical analyses with *p* values smaller than 0.05 were considered to be statistically significant.

## Results

**Fig. 1:**
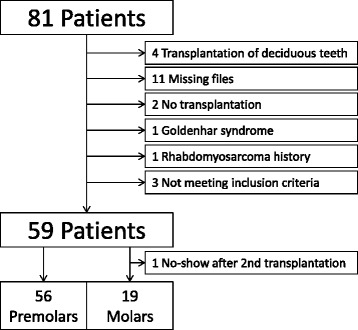
Patient flow chart

**Fig. 2:**
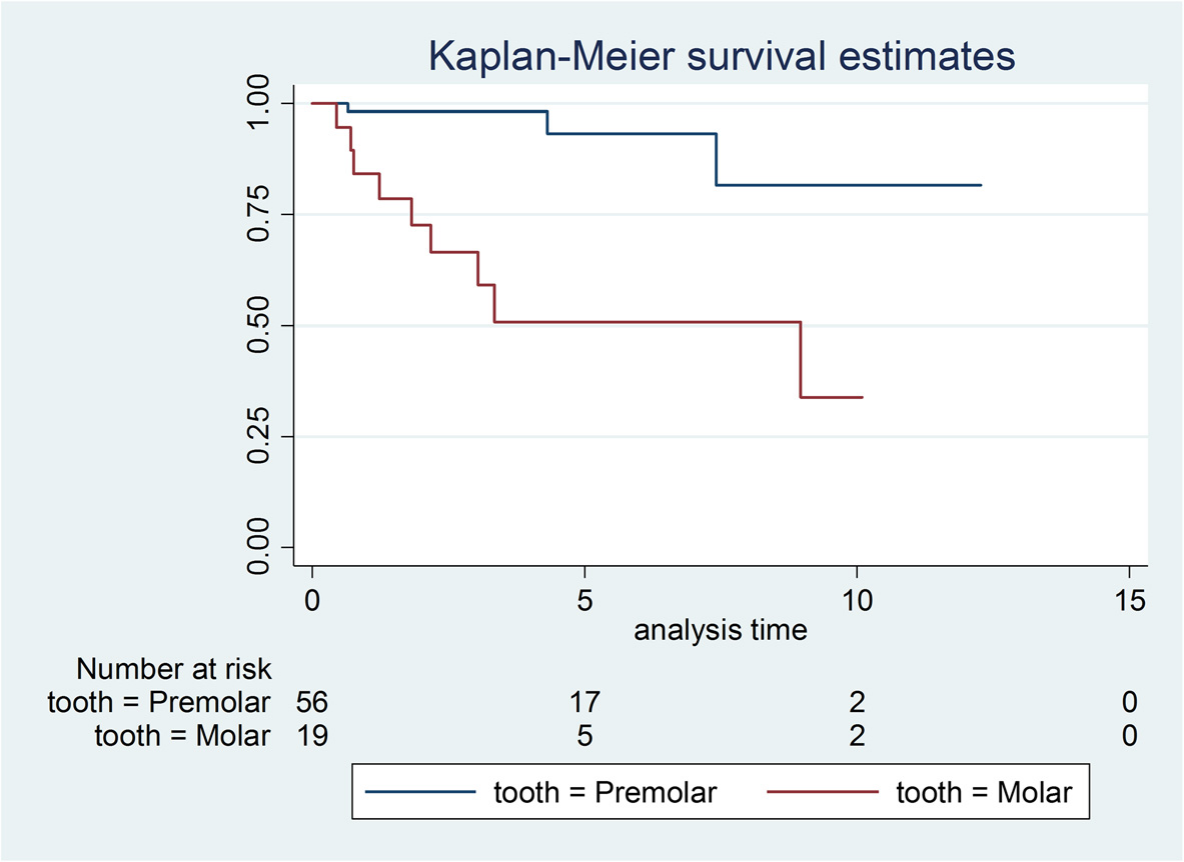
Kaplan-Meier plot comparing success of premolars and molars

**Fig. 3:**
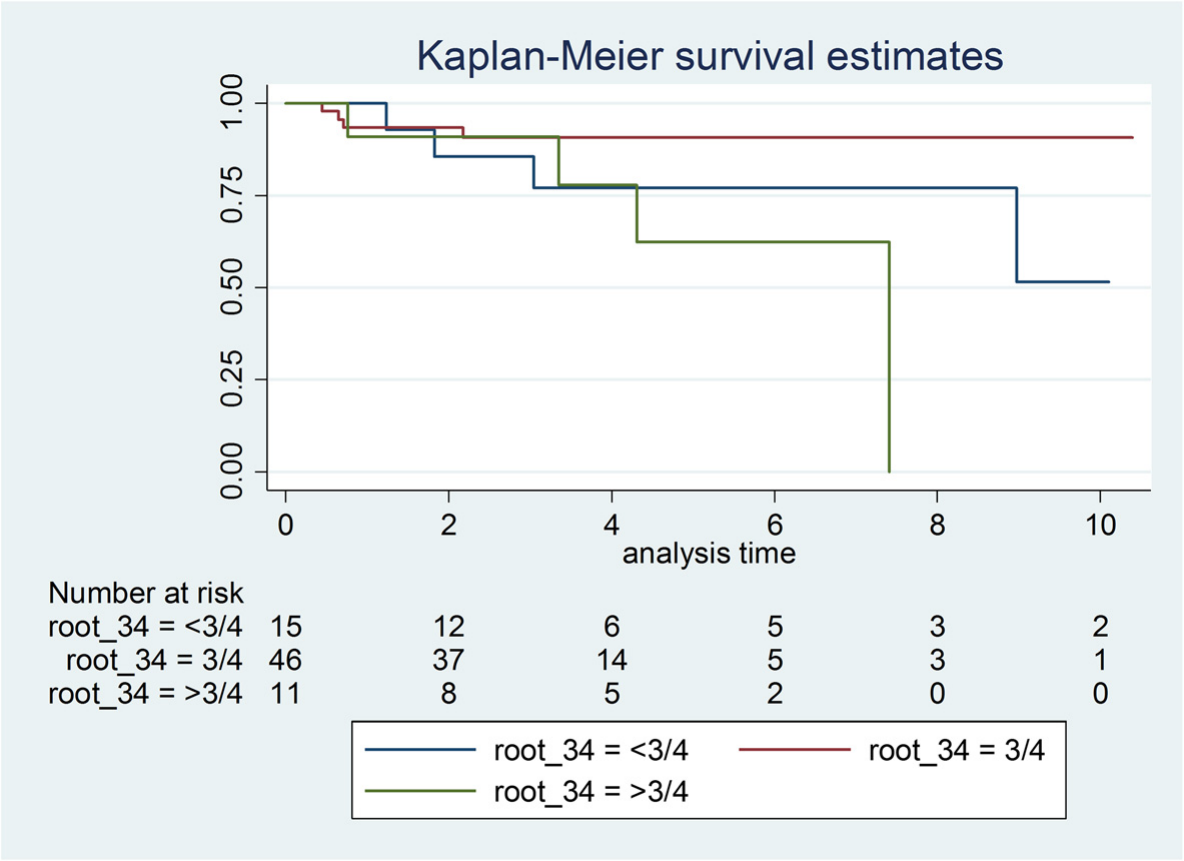
Kaplan-Meier plot comparing success of three grouped root development stages

**Fig. 4:**
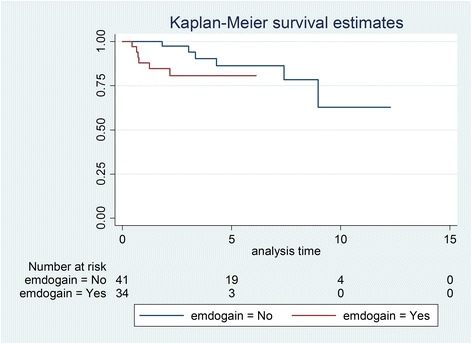
Kaplan-Meier plot comparing success of transplantations with or without use of enamel matrix proteins

**Fig. 5:**
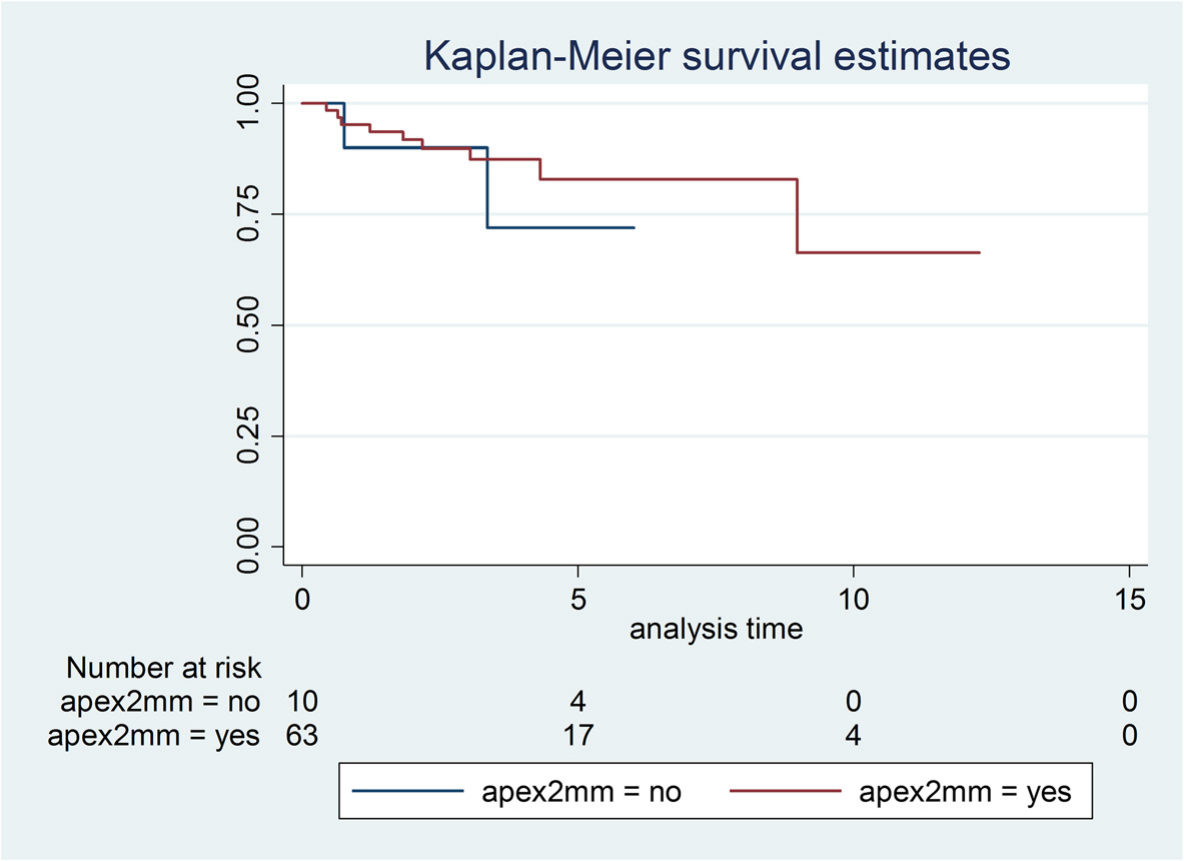
Kaplan-Meier plot comparing success of teeth with apex larger or smaller than 2 mm

**Fig. 6:**
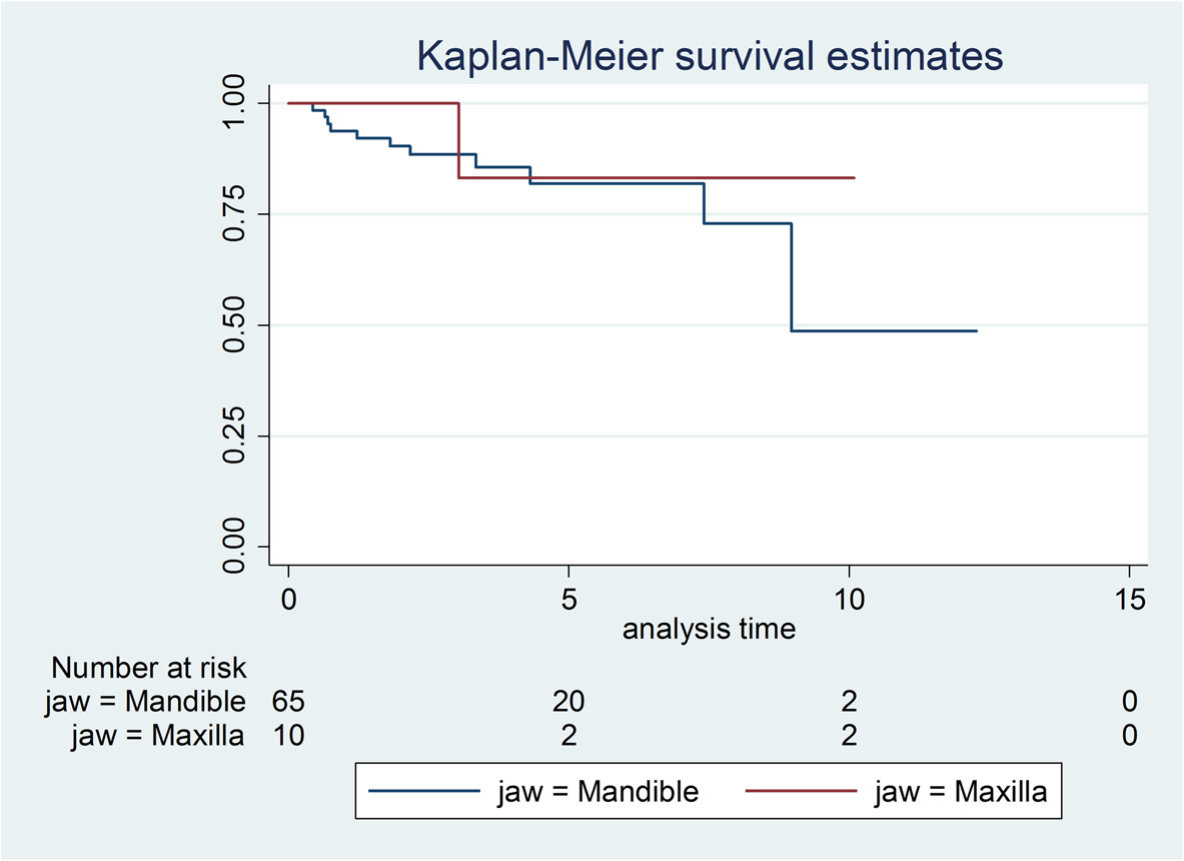
Kaplan-Meier plot comparing success of the recipient sites (mandible/maxilla)

**Fig. 7:**
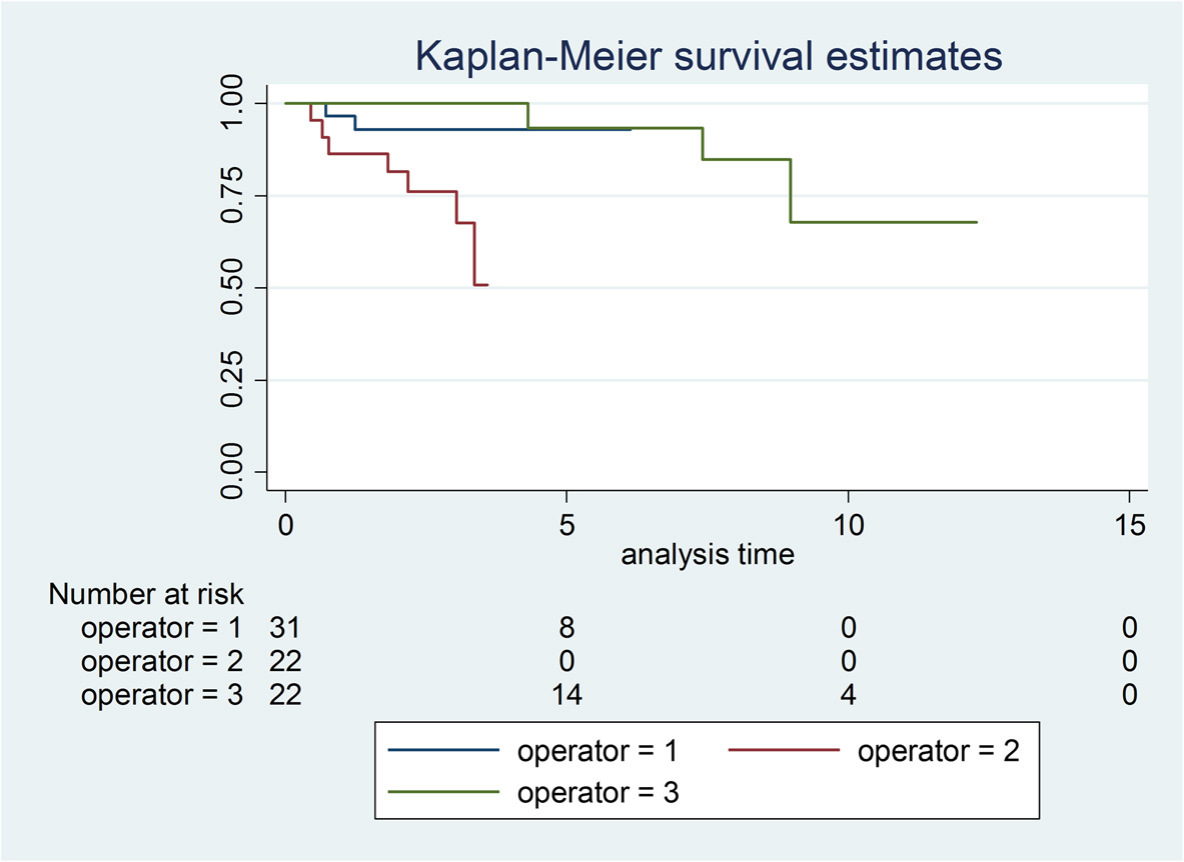
Kaplan-Meier plot comparing success of the three surgeons

Initially, 81 patients were identified, and after exclusions, 59 patients with 75 transplanted teeth (56 premolars, 19 molars) were included. Data of the latter were analysed in this study. Fourteen patients had undergone a transplantation of molars (wisdom teeth) and 53 of premolars. Reasons for drop outs can be seen in Fig. 1. Overall mean age at transplantation was 13.53 years (SD 2.67), the mean age for premolar transplantation being 12.38 years (SD 1.33) and for molars being 16.91 years (SD 2.78). Mean observation time for premolars was 4.06 years (SD 2.44, range 0.64–12.28 years), while for molars the mean was 3.63 years (SD 3.00, range 0.44–10.10 years). Mean observation time for all included teeth was 3.95 years (SD 2.58, range 0.44–12.28 years). Table 1 displays the distribution of transplantations per location, use of enamel matrix proteins and surgeon. Kaplan-Meier cumulative success rate estimation at 10 years for all teeth was 59.6 %. When success was computed for each tooth type separately, premolars showed a 10-year success rate of 81.6 %, whereas molars showed 33.8 % for the same time period, the difference being highly significant (*p* < 0.001) (Fig. 2). No significant evidence of association was observed between success and root development stage (*p* = 0.13), use of enamel matrix proteins (*p* = 0.10), apex width (*p* = 0.59) or the recipient area (*p* = 0.48). However, strong associations between success and surgeon (*p* = 0.001) and success and tooth type (*p* < 0.001) were identified (Table 2). Kaplan-Meier plots for root development, use of enamel matrix proteins, apex width and surgeon are shown in Figs. 3, 4, 5, 6 and 7.

**Table 1:** Distribution of transplantations per location, use of enamel matrix proteins and surgeon

	All teeth (75)	Premolars (56)	Molars (19)
Recipient area			
Maxilla	10 (13.5 %)	5 (8.9 %)	5 (26.3 %)
Mandible	65 (86.7 %)	51 (91.1 %)	14 (73.7 %)
Enamel matrix proteins			
Yes	34 (45.3 %)	27 (48.2 %)	7 (36.8 %)
No	41 (64.7 %)	29 (51.8 %)	12 (63.2 %)
Surgeon			
1	31 (41.3 %)	28 (50.0 %)	3 (15.9 %)
2	22 (29.3 %)	13 (23.2 %)	9 (47.4 %)
3	22 (29.3 %)	15 (26.8 %)	7 (36.8 %)

**Table 2:** Analysis of associations between success and potentially influencing factors using the log-rank test. *p* values <0.05 were considered to be statistically significant

Success	*p* value
Tooth type (premolars/molars)	<0.001
Root development (<3/4, 3/4, >3/4)	0.13
Enamel matrix protein use (yes/no)	0.10
Apex width (>2/<2 mm)	0.59
Recipient area (maxilla/mandible)	0.48
Surgeon (1, 2, 3)	0.001

## Discussion

Our sample showed a vast range of observation periods. This can be explained by the fact that failures, when they happened, often occurred at an early stage after transplantation. In contrary, some teeth had been observed over a long period of time for protocol reasons (consecutive patients). Since only few successful transplantations had observation times shorter than 1 year and by analysing censored data using Kaplan-Meier and log-rank tests with failure as the event, results account for the wide range of observation periods.

Several factors are likely to be associated with survival and success of tooth transplantations. We examined the effect of tooth type, root development stage, recipient area, surgical trauma (surgical skill) and use of intraoperative drugs on success of tooth transplantations.

Premolars with an estimated 10-year success rate of 81.6 % compared favourably to molars with an estimated 10-year success rate of 33.8 %. Thus, while our results for premolars were comparable with most published studies [29], molars in other studies with similar observation periods showed better outcomes [12–14]. However, it should be noted that the first and only failure for molar transplantations conducted by surgeon 3 (most experienced surgeon) occurred after an observation time of 8.97 years while 4 failures out of 9 molar transplantations were seen for surgeon 2 (least experienced surgeon) within a 3.35-year period. Thus, the lower success and survival rates might also reflect the individual surgical skill, learning curve or capacity of case selection of the corresponding surgeon. This interpretation is also underlined by combining the highly significant difference between surgeons (*p* = 0.001) when analysed with the log-rank test (Table 2) with the curves on Fig. 7. While surgeons 1 and 2 (experienced surgeons) seem to perform equally well, surgeon 2 (least experienced surgeon) performed consistently worse. The higher failure rate of molar transplantations compared to premolar transplantations can also be related to more complex root anatomy, potentially leading to more tissue trauma as has been suggested by Denys [15].

The use of enamel matrix proteins did not improve success in our sample. However, because of the small number of events, results should be interpreted with caution. Nevertheless, since prior studies without use of enamel matrix proteins performed equally well for premolars and better than our sample for molars [6, 12, 30], it is still uncertain whether the use of this agent for tooth transplantations is beneficial and the higher cost justified [26, 31–33]. It is also noteworthy to mention that in Fig. 4 cases where the agent was used seemed to perform worse, though no significance was reached. Nevertheless, some investigators have suggested the use of enamel matrix proteins when treating ankylosed teeth [22, 34]. Therefore, the effect of this substance remains uncertain, and large and well-designed randomized clinical trials should be considered.

Our study found no significant associations between success and root development; however, it is a common clinical practice at our clinic to avoid transplantation of teeth with complete root formation, and thus, to reach a significant level of association as shown in two recent studies [15, 28] was difficult. Nevertheless, on the Kaplan-Meier plot, a tendency of root development greater than three fourths for worse success could be seen, though results were not significant (Fig. 3).

### Limitations

Since the study was retrospective, it is susceptible to limitations interconnected with this type of investigation. By defining endodontic treatment as not being a failure, a potential bias may arise since endodontically treated teeth can develop complications which can lead to necessity for extraction or such rated as failure according to the applied grading system. Nevertheless, in a study by Andreasen [6], teeth receiving endodontic treatment within 4 weeks from transplantation showed a 4-year survival rate of 95 %; therefore, endodontic treatment was not judged as a failure. We made an effort to include all cases to minimize selection bias; however, confounding was difficult to adjust for the given small number of events which gave unstable results in the multivariable analyses that were omitted. The sample size especially for molars and the number of events were small which suggests caution when interpreting the results. However, this exploratory study has value in terms of suggesting areas of focus for future studies.

## Conclusions

Molar transplantations were not as successful as premolar transplantations; however, success rates also varied greatly depending on the surgeon’s experience. In our sample, the use of enamel matrix proteins as well as root development stage, the recipient area and apex width did not show significant associations with success of tooth transplantations.
